# Influence of HFCVD Parameters on Diamond Coatings and Process Investigation of Sapphire Wafer Lapping

**DOI:** 10.3390/ma19030584

**Published:** 2026-02-03

**Authors:** Wei Feng, Shuai Zhou, Xiaokang Sun

**Affiliations:** School of Mechanical Engineering, Yancheng Institute of Technology, Yancheng 224001, China; zhoushuai@stu.ycit.edu.cn (S.Z.); sunxiaokang@stu.ycit.edu (X.S.)

**Keywords:** HFCVD, diamond film, sapphire wafer, lapping

## Abstract

Aiming at the key problems of the material removal rate and surface integrity of existing tools in the lapping of sapphire hard and brittle crystals, an efficient lapping tool has been developed to explore a new process for HFVCD (hot filament chemical vapor deposition) diamond tools to efficiently lap sapphire wafers. With the premise of ensuring the surface roughness of the wafer is Ra ≤ 0.5 μm, the material removal rate is increased to more than 1 μm/h. To explore a high-efficiency lapping process for sapphire wafers using HFCVD diamond tools. The influence of key preparation parameters on the surface characteristics of CVD (chemical vapor deposition) diamond films was systematically investigated. Three types of CVD diamond coating tools with distinct surface morphologies were fabricated. These tools were subsequently employed to conduct lapping experiments on sapphire wafers in order to evaluate their processing performance. The experimental results demonstrate that the gas pressure, methane concentration, and substrate temperature collectively influenced the surface morphology of the diamond coatings. The fabricated coatings exhibited well-defined grain boundaries and displayed pyramidal, prismatic and spherical features, corresponding to high-quality microcrystalline and nanocrystalline diamond layers. In the lapping experiments, the prismatic CVD diamond coating tool exhibited the highest material removal rate, reaching approximately 1.7 μm/min once stabilized. The spherical diamond coating tool produced the lowest surface roughness on the lapped sapphire wafers, with a value of about 0.35 μm. Surface morphology-controllable diamond tools were used for the lapping processing of the sapphire wafers. This achieved a good surface quality and high removal rate and provided new ideas for the precision machining of brittle hard materials in the plane or even in the curved surface.

## 1. Introduction

Chemical vapor deposition (CVD) diamond films possess hardness, thermal conductivity, and elastic modulus values that have reached or are approaching those of the natural diamond. In addition, CVD diamond films offer advantages such as geometric flexibility, strong impact resistance, and favorable self-lubricating behavior. These attributes endow them with broader application potential compared with conventional diamond grains [1,2,3]. At present, among the diverse applications of CVD diamond films, their use as coatings for cutting tools has become particularly widespread. The exceptionally high hardness and low friction coefficient of diamond films render them outstanding candidates for advanced tooling applications [4,5,6]. As coatings for diamond cutting tools, CVD diamond layers typically exhibit thicknesses ranging from several micrometers to several tens of micrometers. The substrates used for coated tools are usually selected from materials that offer ease of fabrication, since such substrates can be manufactured into complex geometries and are therefore suitable for tools with intricate shapes. CVD diamond coatings offer short deposition times and relatively low fabrication costs.

Compared with other types of diamond tools, such as PCD (polycrystalline diamond) tools and brazed diamond tools, they present significant advantages in manufacturing efficiency, resource utilization, diversity of surface morphologies, overall cost, and suitability for tools with complex geometries. Specifically, the CVD-coated tool can coat hundreds of tool substrates with arbitrary shapes simultaneously in one batch, and the deposition process can operate continuously for 24 h without a cooling wait cycle. In contrast. PCD tools need to go through the process of “mixing diamond micro powder metal binder–pressing–high temperature and high pressure sintering at 1400 °C–cooling–follow-up machining”. The overall manufacturing cycle of PCD tools is three to five times that of CVD of the same type [7,8,9]. In order to ensure the strength of the tool, 20–40 vol% of diamond micro powder needs to be added the PCD sintered body, and only 40–60% of the diamond can participate in the actual process. Joshi et al.’s research shows that the diamond resource consumption of PCD tools is 500–1000 times that of CVD tools under the same machining life, and this resource waste will directly push up the processing cost in the processing of precious and brittle materials [10]. The CVD process only produces a small number of hydrocarbon by-products, which can be discharged up to the standard after treatment. However, the PCD sintering process consumes a large amount of energy, and the use of a metal binder (such as Co) may bring potential environmental risks. The brazed diamond tool needs to use brazing flux in the braz process, and the discharge of a large amount of sewage is caused by cleaning the residual brazing flux. So, the CVD diamond tool is superior to PCD and braz-diamond tools in terms of resource conservation and environmental friendliness [11]. Junior et al. prepared boron-doped grooved textured diamond coating on the tool surface by laser structuring combined with HFCVD technology, which reduced the friction coefficient by 40%, increased the bonding strength by 50%, and reduced the wear diameter of the counterpart by 16.7%. This functional customization capability makes CVD tools adapt to the multi-process processing requirements of “rough cutting–fine grinding–polishing” [12]. CVD diamond coating is a pure polycrystalline diamond structure metal binder, and it has excellent heat stability. In the high-speed milling test of high-performance isotropic graphite, the wear of the rear rake face of the CVD-composite-coated tool was only 0.25 mm after 45 min of machining, which did not reach the standard of bluntness (0.3 mm). The wear of the rear rake face of the PCD tool exceeded 0.3 mm after 25 min of machining. The service life of the CVD tool was more than 1.8 times that of the PCD tool [13]. Heaney et al.’s research shows that when processing aluminum alloys, the cutting force CVD-coated micro-end mill (diameter 300 μm) is reduced by 50%, and the machining accuracy can reach ±1 m when processing aluminum alloys, which can meet the machining requirements of MEMS devices and microfluidic devices [14]. In the curved surface machining of hard and brittle crystalline materials such as gallium oxide and sapphire, CVD-coated tools achieve high-precision machining of complex curves through curved substrate coating, and the machining efficiency is increased by more than 50% compared with PCD tools; thus, the problem of particle shedding in the curved surface machining of PCD tools is avoided. Thus, it can be seen that the CVD diamond method has significant advantages in many aspects of machining of workpieces.

Among the various methods for diamond synthesis, the hot-filament chemical vapor deposition (HFCVD) technique has been the most extensively investigated for producing diamond films [15,16,17,18]. This method is widely recognized due to its simple equipment configuration, ease of parameter control, stable growth behavior, and suitability for scaling to large deposition areas and batch production, which has led to its broad acceptance and application [19,20]. In studies concerning HFCVD diamond films, their friction and wear behavior has received the greatest attention, since it directly determines the ultimate performance and service life of the films. Kumar et al. fabricated nanocrystalline diamond films and investigated their super lubricity characteristics. Under low load, vacuum, or inert atmosphere, the friction coefficient can be stably maintained in the super-lubricating range (<0.01), and the wear rate is extremely low, combining the dual advantages of high hardness and super-lubrication [21]. Zhu et al. conducted a systematic study on the polish ability and tribological performance of various types of CVD diamond coatings. The boron-doped nanocrystalline coating shows the best performance among all the coatings in the water lubrication environment, with a stable friction coefficient between 0.03 and 0.05, and a low wear rate of 1.2 × 10^−7^ mm^3^/(N·m). In the dry friction condition, the friction coefficient of all kinds of coating increases, and the nanocrystalline coating has better wear resistance than the microcrystalline coating [22]. Shen et al. modified the deposition parameters to grow single-layer microcrystalline diamond, single-layer submicrocrystalline diamond, and multi-layer microcrystalline diamond films on Si_3_N_4_ and cemented carbide substrates. The polycrystalline microcrystalline diamond films were formed through successive stacking of diamond grains, resulting in a densely packed structure [23]. Nanocrystalline diamond layers can be deposited onto tools with relatively small radii, and their high density of grain boundaries imparts considerable fracture strength. Their electrical, thermal, and optical properties can vary across several orders of magnitude, enabling favorable electrical conductivity even at ambient conditions. Furthermore, modification of the surface chemistry of diamond films can impart hydrophilic characteristics. Zheng et al. examined the influence of different interlayer materials and deposition conditions on the tribological performance of diamond films. When the substrate roughness was 0.6 μm, the deposited diamond film exhibited the lowest friction coefficient and wear rate, corresponding to the optimal wear resistance [24].

Overall, previous studies indicate that a detailed investigation of the effects of deposition parameters on diamond film properties remains essential. Moreover, the capability of depositing diamond films onto substrates with diverse geometries, including spherical surfaces, endows the resulting tools with excellent fracture strength as well as strong resistance to acidic and alkaline corrosion. Building upon this foundation, further investigation into the surface morphology and grain arrangement of diamond film tools is required to achieve controlled microstructural features. Sapphire (α-Al_2_O_3_) substrates are single-crystal materials with excellent physical and chemical properties, good thermal conductivity, superior chemical stability, and high transmittance in the ultraviolet (UV) to infrared (IR) range. These characteristics make them ideal for applications in optoelectronics, semiconductor power devices, and precision optics. However, sapphire is a typical hard and brittle material. Conventional machining processes will inevitably leave sub-surface damage layers and residual stress on the substrate surface. Polishing is thus a critical ultra-precision machining step to eliminate these defects and achieve atomically flat surfaces. The innovative application of CVD diamond film tools in the lapping of sapphire domes offers the potential to elucidate the lapping characteristics and processing mechanisms associated with this method. Such advancements are expected to exert a profound and transformative influence on the precision finishing of sapphire domes.

## 2. Experiment Methods

### 2.1. Deposition of Diamond Coatings

**Figure 1:**
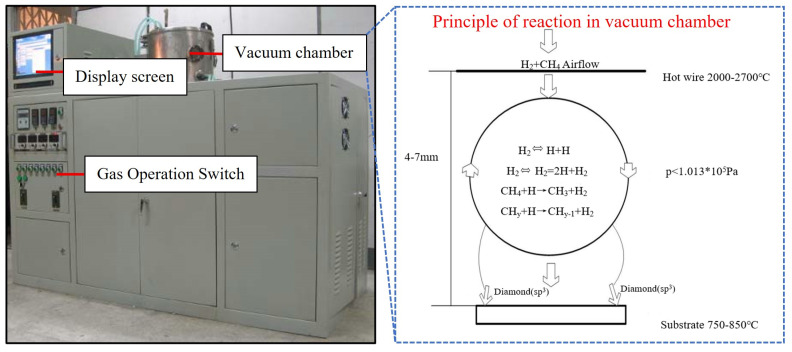
Deposition equipment configuration.

A compact HFCVD system was employed to deposit the diamond coatings, and the Laboratory made equipment configuration is illustrated in Figure 1. Figure 1 also shows the chemical equation that forms the basis of the CVD method. The high-temperature radiation from the hot filament in an HFCVD system can induce homolytic cleavage of hydrogen molecules, generating highly reactive hydrogen radicals. Methane is cracked into methyl radicals and hydrogen radicals, which are eventually converted into active carbon atoms. A selective etching reaction takes place in the system: high concentrations of hydrogen radicals preferentially combine with sp^2^-hybridized graphite-phase carbon, converting it back into gaseous methane that desorbs from the substrate. In contrast, sp^3^-hybridized diamond-phase carbon, owing to its high bond energy, is not susceptible to etching by hydrogen radicals, thereby facilitating the formation of diamond.

The substrate specimens were cemented carbide (Zhuzhou Chaoyu Industrial Co., Ltd., Zhuzhou, China), consisting of 6 percent Co and 94 percent WC, with dimensions of 3 mm × 6 mm × 1.5 mm. Tungsten filaments with a diameter of 40 μm were selected as the heating elements, and CH_4_ and H_2_ were used as the reactive gas sources. Five tungsten filaments were arranged in a parallel and uniformly spaced configuration above the substrate, using a supporting structure, with a separation distance of approximately 6–7 mm from the substrate surface to ensure a uniform temperature field across the substrate area. Prior to the deposition of diamond, newly installed tungsten filaments were subjected to a carburization process. The carburization typically lasted for approximately 40 min and continued until the electrical resistance of the filaments ceased to increase significantly. The deposition parameters used for preparing the coatings are summarized in Table 1. After deposition, the resulting CVD diamond samples were characterized using an S3400 scanning electron microscope (Hitachi High-Tech Corporation, Tokyo, Japan), a D8 Advance X-ray (Bruker with a diffraction angle range of 25°~125°, Billerica, MA, USA) and a laser confocal micro-Raman spectrometer operating at a wavelength of 514.5 nm.

### 2.2. Lapping Experiments on Sapphire Wafers Using Diamond-Coated Tools

**Figure 2:**
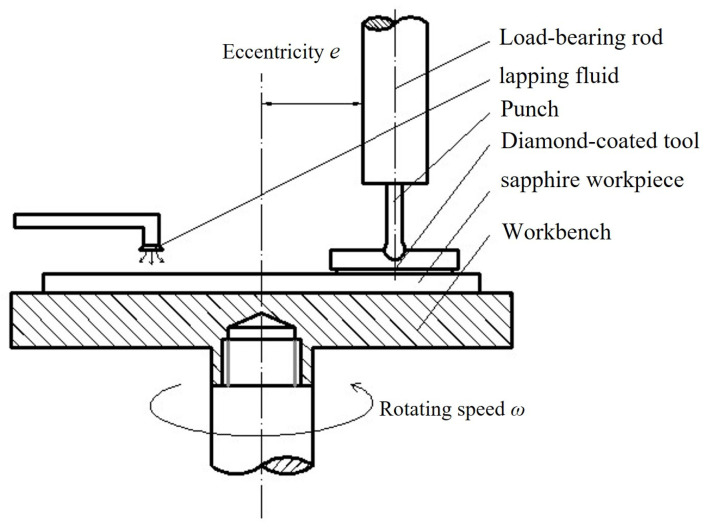
Schematic diagram of the lapping principle of the self-made diamond-coated tool grinding test machine.

Under ambient laboratory conditions of 20–25 °C, the sapphire substrates were lapped using the CVD diamond-coated tools. The operating principle of the experimental apparatus is illustrated in Figure 2. A normal load of 2 N was applied, with a rotational speed of 50 r/min and an eccentricity of 10 mm. The lapping fluid consisted of deionized water and 3% triethanolamine. The variations in friction force and the wear behavior of sapphire substrates in contact with CVD diamond coatings of different surface morphologies were examined. The wear track depth was measured using a KLA-Tencor profilometer, and the surface roughness was characterized with a NanoMap-500LS three-dimensional surface profiler. The material removal rate (MRR) was quantified as the reduction in the thickness of the sapphire wafer per unit time. It was determined by measuring the cross-sectional profiles of the wear tracks, using the profilometer, and calculating the average depth increase divided by the lapping duration. For each substrate, measurements were taken at 5~6 locations and averaged. The experimental specimens were single-crystal sapphire wafers supplied by Changzhou Zhongjing Technology Co. (Changzhou, China), with the {0001} crystallographic plane as the processed surface. The wafers had a diameter of 50.8 mm, an average initial thickness of 0.6 mm, and an initial surface roughness, Ra, of approximately 0.64 μm.

## 3. Results and Discussion

### 3.1. Analysis of Diamond Coatings

#### 3.1.1. Effect of Reaction Pressure

**Figure 3:**
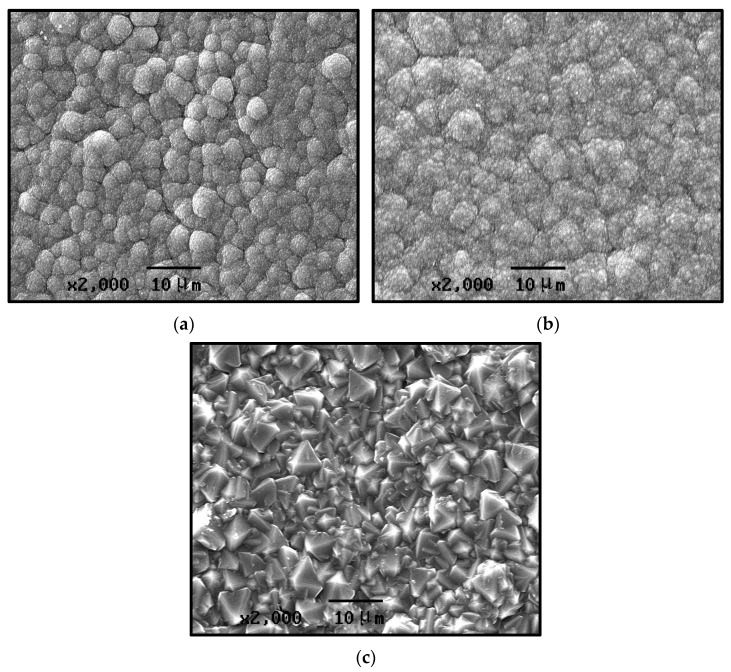
SEM images of CVD diamond-coated growth surface at different gas pressures. (**a**) 1 kPa; (**b**) 2 kPa; and (**c**) 3 kPa.

Figure 3 presents SEM images of the growth surface of CVD diamond films, deposited under different reaction pressures. For the sample prepared at a reaction pressure of 1 kPa, pronounced grain agglomeration is observed, with the crystallites clustering into spherical aggregates. The individual grains are extremely fine, making their shapes difficult to distinguish; however, the agglomerated particles exhibit a generally uniform size (Figure 3a). For the sample prepared at a reaction pressure of 2 kPa, the diamond film exhibits poor crystallinity, with grain sizes ranging from several tens to several hundreds of nanometers. Grain agglomeration is evident, and although the grains show a clear tendency to increase in size, the overall uniformity of the film deteriorates (Figure 3b). The crystallite shape clarity and integrity are relatively low, and pronounced secondary nucleation can be observed. The individual crystal facets and grain boundaries are largely indistinguishable. Figure 3c shows the sample prepared at a reaction pressure of 3 kPa, which exhibits a typical microcrystalline diamond morphology. The grain size is relatively uniform, predominantly ranging from 2~5 μm. The grains are densely and uniformly packed, and secondary nucleation phenomena are not evident. Due to the laboratory-scale HFCVD growth equipment we use, a large number of experiments have shown that the suitable gas pressure parameter range is 1–3 Kpa. Excessively high pressure disrupts the balance between the generation and the transport of active species, nucleation-growth coordination, and thermodynamic stability required for diamond growth, leading to the failure of the synthesized diamond surface to exhibit distinct morphological characteristics.

From the first set of experimental conditions in Table 1 (Numbers 1, 2, and 3), it can be concluded that the deposition pressure in the HFCVD process has a significant influence on the surface morphology of the diamond coatings. At relatively low pressures, the growth surface exhibits much smaller grains with indistinct grain boundaries, accompanied by a pronounced secondary nucleation phenomenon. The grain size of the diamond increases progressively with the rising deposition pressure, reaching the micrometer scale at higher pressures. Under these conditions, the grain boundaries become well-defined and secondary nucleation phenomena disappear.

This behavior is primarily attributed to the variation in the concentration of thermally dissociated active species (such as H and CH_3_ radicals) at different reaction pressures, rather than changes in kinetic energy [25]. At relatively low reaction pressures (e.g., 1 kPa), the absolute concentration of atomic hydrogen in the gas phase is low. Consequently, the etching efficiency of atomic hydrogen on non-diamond carbon phases (sp2-bonded carbon) and secondary nuclei is reduced. The accumulation of unetched amorphous carbon and surface defects provides numerous active sites for renucleation, thereby leading to a high rate of secondary nucleation and the formation of fine-grained, cauliflower-like agglomerates. Conversely, with increasing reaction pressure, the concentration of atomic hydrogen rises significantly. The enhanced presence of atomic hydrogen promotes the selective etching of secondary nuclei and non-diamond carbon deposits. This strong etching effect effectively suppresses secondary nucleation, creating a clean growth environment that favors the continuous enlargement of primary crystallites. As a result, the grain size increases, and the crystals exhibit well-defined facets and boundaries, as observed in the sample deposited at 3 kPa.

#### 3.1.2. Effect of Methane Concentration

**Figure 4:**
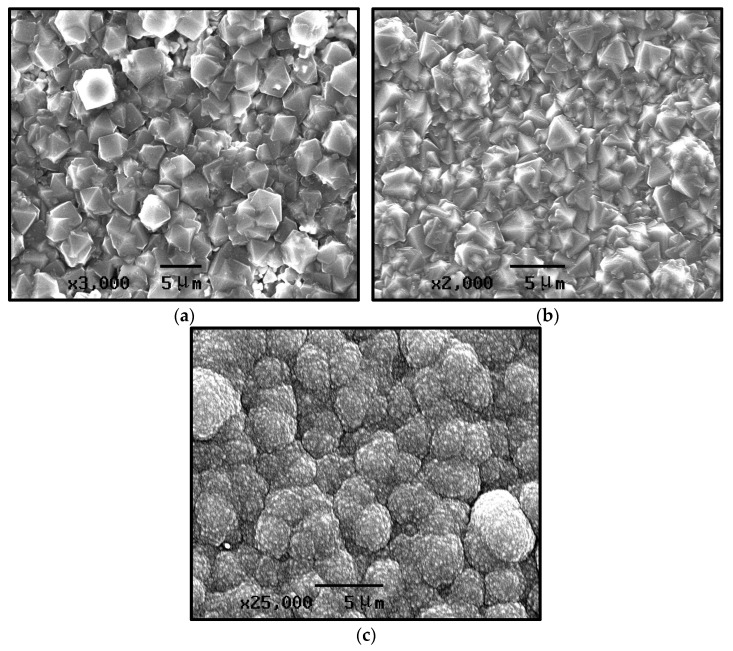
SEM images of CVD diamond-coated at different methane concentrations. (**a**) 1%; (**b**) 2%; and (**c**) 3%.

Figure 4 presents SEM images of the growth surfaces of CVD diamond films prepared under different methane concentrations. Figure 4a shows the sample deposited at a methane concentration of 1%, which displays a typical microcrystalline diamond morphology. The grain size reaches the micrometer scale and is relatively uniform, with an average diameter of approximately 5 μm. The grains are tightly bonded to one another, with no evidence of secondary nucleation. Figure 4b shows the sample prepared at a methane concentration of 2%. The grain diameter is approximately 5 μm, which is indicative of a microcrystalline diamond morphology. However, fine secondary nucleation can be observed along the grain boundaries, where the small crystallites exhibit a noticeable variation in size.

Figure 4c shows the sample prepared at a methane concentration of 3%. The nucleation density of the diamond increases markedly, and secondary nucleation becomes pronounced. The crystallinity of the grains is relatively low, and grain agglomeration occurs, forming coarse spherical aggregates. Numerous voids are generated between these clustered particles, and the grain boundaries become indistinct. The sizes of the agglomerated spherical clusters vary considerably, with the largest reaching approximately 5 μm.

From the first set of experimental conditions in Table 1 (Numbers 4, 5, and 6), it can be concluded that under the present deposition parameters, the grain size decreases progressively with an increasing methane concentration. When the concentration reaches 3%, secondary nucleation becomes pronounced, and the grains agglomerate to form coarse spherical clusters. This is primarily attributed to the relatively high concentration of atomic hydrogen in the gas phase when the carbon source concentration is low. Under these conditions, carbon-containing species such as CH_3_^−^ and CH_2_^2−^ undergo dehydrogenation reactions induced by atomic hydrogen, leading to the formation of diamond through sp^3^ bonding. At the same time, atomic hydrogen etches the graphitic phase, causing its gasification. As a result, the quality of the diamond is relatively high, exhibiting well-defined grain boundaries and sharply faceted crystallites. However, atomic hydrogen also exerts a certain etching effect on the diamond phase itself, and its etching efficiency varies markedly among diamond crystal planes with different crystallographic orientations. In contrast, the higher surface densities of non-(100) planes lead to more pronounced etching. The formation of the (100) texture is governed by the evolutionary selection rule (Van der Drift model) [26]. A high atomic hydrogen concentration effectively suppresses secondary nucleation by selectively etching $sp^{2}$ bonded non-diamond carbon phases. Furthermore, under these conditions, the growth parameter α ($\alpha =\sqrt{3}\cdot V_{100}/V_{111}$) changes such that the growth rate [27] of the (100) faces becomes the slowest, relative to other crystallographic directions. Consequently, the faster-growing (111) facets grow out of existence, leaving the thermodynamically stable (100) facets as the dominant surface morphology. Recent atomic-scale investigations confirmed that the (100) surface exhibits superior thermal stability compared to the (111) surface [28].

Consequently, when the concentration of atomic hydrogen is relatively high, a (100) texture develops under the specific conditions of this experiment. Under the condition of elevated carbon source concentration, the gas phase contains a higher abundance of carbon-containing reactive species, which increases the rate of secondary nucleation. At the same time, the relative decrease in atomic hydrogen concentration reduces its ability to etch the non-diamond carbon generated on the diamond surface. As a result, the overall quality of the diamond deteriorates, leading to blurred crystal facets and reduced grain integrity.

#### 3.1.3. Effect of Substrate Temperature

**Figure 5:**
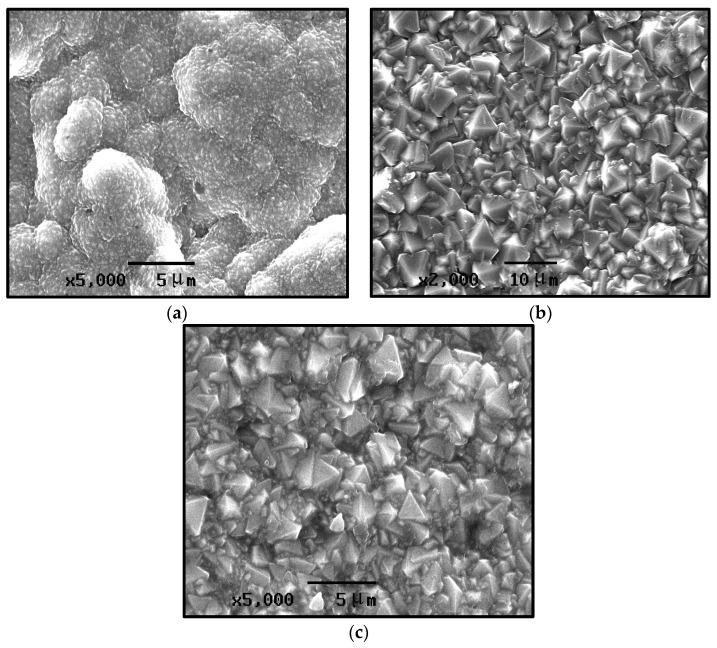
SEM images of CVD diamond-coated at different substrate temperatures. At (**a**) 730 °C; (**b**) 780 °C; and (**c**) 830 °C.

Figure 5 shows SEM images of the growth surfaces of CVD diamond coatings deposited at different substrate temperatures, illustrating the influence of substrate temperature on the surface morphology. Figure 5a corresponds to the sample prepared at a substrate temperature of 730 °C. Under these conditions, the diamond grains are extremely fine, with indistinct crystal facets and poorly defined grain boundaries, and the overall morphology exhibits a cauliflower-like appearance. Figure 5b shows the sample deposited at a substrate temperature of 780 °C. Compared with Figure 5a, the individual diamond grains become larger, the surface becomes rougher, and the grain boundaries appear to be well-defined with clearly faceted features. Figure 5c corresponds to the sample prepared at a substrate temperature of 830 °C. Relative to Figure 5b, the grain size generally falls within the range of 1~3 μm.

The SEM images of diamond growth surfaces obtained at different substrate temperatures indicate that the substrate temperature exerts a noticeable influence on the surface morphology of the diamond coatings. Within the substrate temperature range of 730~780 °C, the surface morphology of the CVD diamond coatings changes substantially as the temperature increases. In this interval, the cauliflower-like agglomerates on the coating surface gradually evolve into sharply faceted, typical micrometer-sized diamond grains. At relatively low deposition temperatures, fewer active hydrocarbon species are adsorbed on the substrate surface, and these species possess lower free energy. As a result, the nucleation energy for diamond formation becomes insufficient, leading to a low nucleation density. In addition, the etching effect of atomic hydrogen on the graphitic phase is weakened at lower temperatures, which promotes the formation of amorphous carbon and graphitic phases. At lower substrate temperatures, the crystal tends toward its equilibrium morphology, favoring octahedral shapes with relatively low surface energy. As the deposition temperature increases, the energy of the active species rises and the growth-rate parameter gradually decreases α ($\alpha =\sqrt{3}V_{100}/V_{111}$) and increases with the substrate temperature. According to the Van der Drift evolutionary selection model, the change in α makes the <100> direction the direction of fastest vertical growth. Consequently, grains oriented with their <100> axes perpendicular to the substrate survive the competitive growth, leading to the development of a <100> texture and the dominance of {100} facets on the film surface [26].

#### 3.1.4. Surface Analysis of Diamond Coatings with Three Distinct Morphologies

Based on the previously established relationships between key processing parameters and the resulting surface morphology of CVD diamond coatings, the experimental conditions were further adjusted to obtain three surface morphologies that were distinct, clearly discernible, and representative of typical structural features. These three characteristic diamond films were subsequently characterized using SEM to examine their surface morphologies.

**Figure 6:**
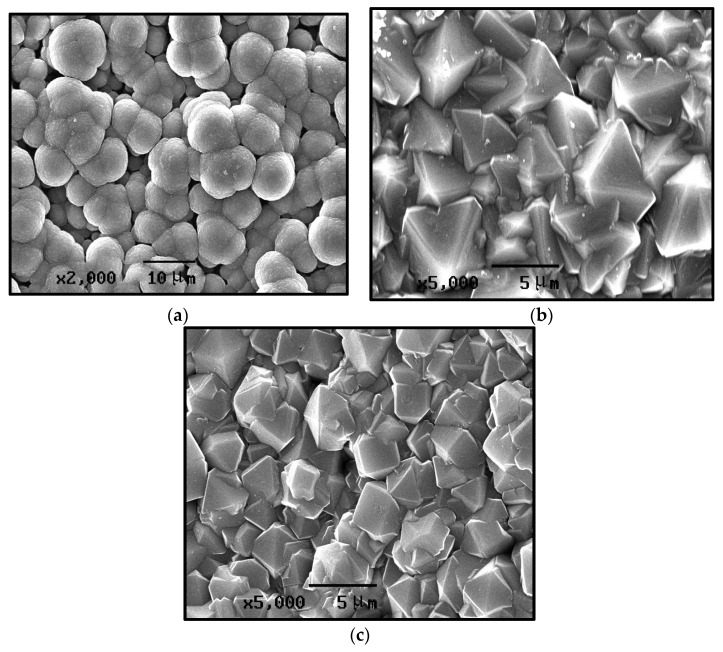
SEM image of the surface of diamond-coated CVD. (**a**) Spherical CVD diamond coating; (**b**) pyramidal CVD diamond coating; and (**c**) prismatic CVD diamond coating.

**Figure 7:**
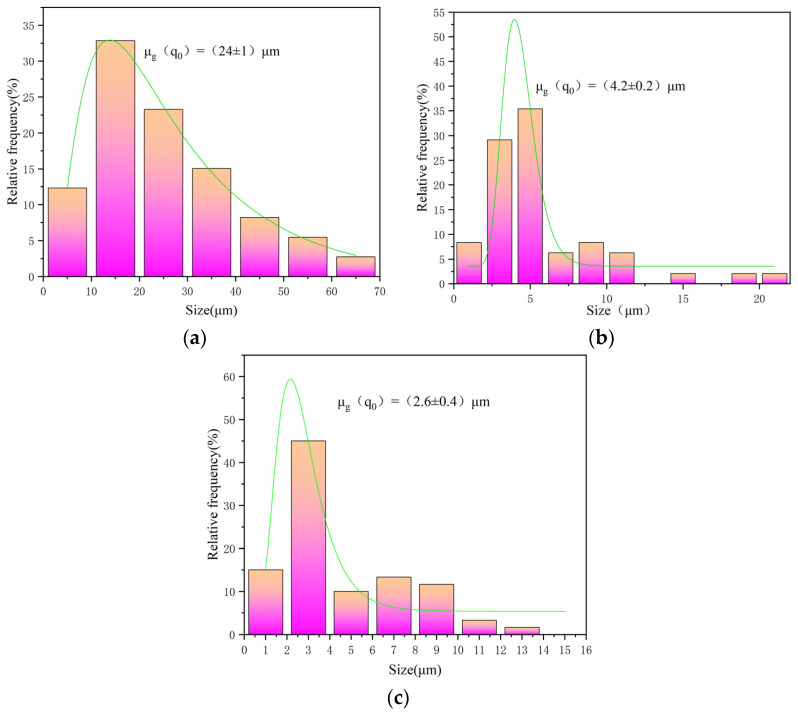
Statistical grain size distributions of the three typical diamond coatings. (**a**) Spherical CVD diamond coating; (**b**) pyramidal CVD diamond coating; and (**c**) prismatic CVD diamond coating.

**Figure 8:**
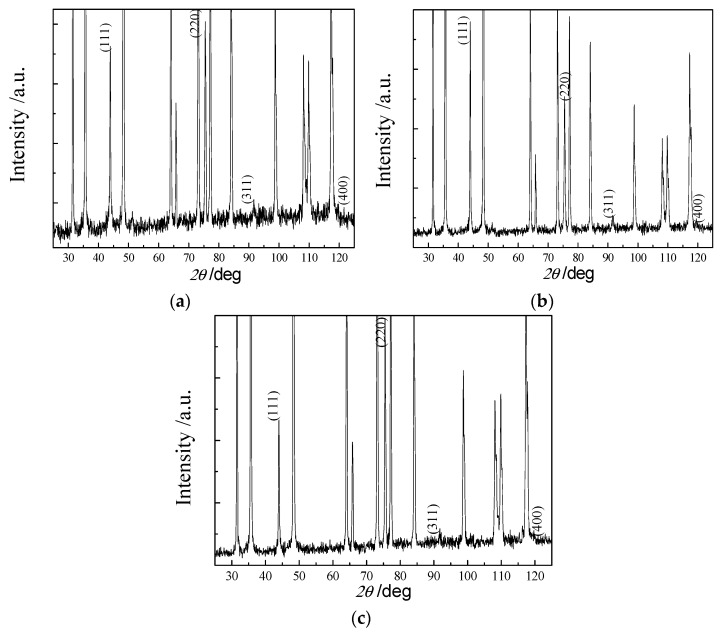
X-ray diffraction spectrum of the surface of diamond-coated CVD. (**a**) Spherical CVD diamond coating; (**b**) pyramidal CVD diamond coating; and (**c**) prismatic CVD diamond coating.

As shown in the SEM image in Figure 6a and grain size distributions in Figure 7a, the surface of the spherical CVD diamond film is composed of globular particles formed by clusters of numerous nanoscale crystallites. The globular particles are cauliflower-like. The individual spherical aggregates exhibit average diameters of approximately 25 μm, whereas the constituent nanocrystallites are significantly smaller in comparison. The XRD pattern indicates the presence of the pronounced diamond and WC phases in this sample. The XRD spectrum of the spherical diamond coating, shown in Figure 8a, displays clear (111) and (220) diffraction peaks, which are typical characteristic peaks corresponding to the cubic syngony of diamond, along with a slight fluctuation near the (311) reflection.

The (111) diffraction peak exhibits significant broadening. Since the tiny nanocrystallite grains of the spherical diamond coating were difficult to measure, the average crystallite size (D) was calculated using the Scherrer equation [29]:

$$D=\frac{K\lambda }{\beta \cos\theta }$$

where K is the shape factor (typically 0.9), λ is the X-ray wavelength (0.15406 nm for Cu K α), β is the full width at half maximum (FWHM) in radians, and θ is the Bragg angle. The calculated average nanoscale crystallite size is approximately 21.4 nm. It is important to note that this value represents an effective crystallite size, as the observed peak broadening is a convolution of both grain size reduction and lattice microstrain induced by the high density of grain boundaries and point defects in the nanocrystalline film.

As shown in Figure 6b, the pyramidal CVD diamond coating exhibits an overall morphology characterized by outward-facing pyramidal facets. The pyramids are relatively uniformly distributed, and after a deposition time of 5 h, the grain size reaches approximately 4 μm (as shown in Figure 7b). The XRD pattern of the pyramidal diamond coating, shown in Figure 8b, reveals distinct diamond (111) and (220) diffraction peaks in addition to the tungsten carbide peaks originating from the substrate. The (111) diffraction peak is exceptionally sharp, with an intensity far exceeding that of the (220) reflection, whereas the (400) peak appears to be relatively weak.

As shown in Figure 6c, the prismatic CVD diamond coating exhibits a surface morphology in which the planar facets are oriented outward. The grain shapes are well-defined, with clearly distinguishable grain boundaries. After a deposition time of 5 h, the grain size reaches approximately 2–3 μm. The grain size distribution is shown in Figure 7c. The XRD pattern of the prismatic coating (Figure 8c) shows a distinct deviation from the random powder diffraction pattern. To quantitatively analyze the preferred orientation, the texture coefficients (TC) were calculated using the Harris method [30]:

$$TC\left(hkl\right)=\frac{I\left(hkl\right)/I_{0}\left(hkl\right)}{\frac{1}{n}\sum_{i=1}^{n}\left[I\left(h_{i}k_{i}l_{i}\right)/I_{0}\left(h_{i}k_{i}l_{i}\right)\right]}$$

where $I\left(hkl\right)$ is the measured relative intensity, $I_{0}\left(hkl\right)$ is the standard intensity from the JCPDS card, and n is the number of diffraction peaks considered. The calculated $TC\left(220\right)$ and $TC\left(400\right)$ values are significantly greater than unity (TC > 1), confirming the presence of a strong <110> and <100> texture. This crystallographic texture supports the SEM observations (Figure 6c), where the film surface is dominated by square-shaped {100} facets and prismatic features, which are consistent with the evolutionary selection of grains under the specific deposition conditions.

**Figure 9:**
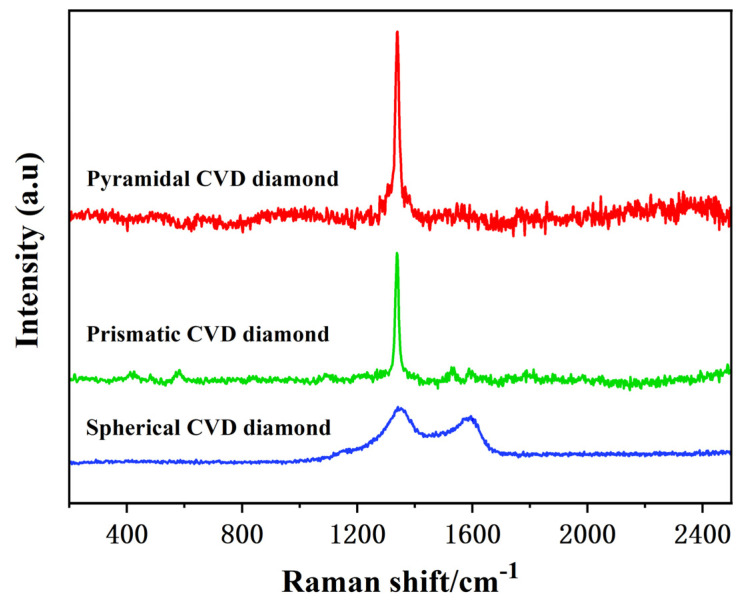
Raman spectrum of the surface of diamond-coated CVD.

The Raman spectrum is shown in Figure 9. The peak at 1332 ± 2 cm^−1^ is the strongest for both the prism and the pyramid coating, which is a typical characteristic of the SP^3^ bond in diamond. The Raman spectrum at about 1330 cm^−1^ can be used as a criterion for evaluating the quality of the diamond film, so the microcrystalline diamond film will appear at this point with a strong peak under certain parameter conditions. It means that the sample grows a microcrystalline diamond film, and the film quality is good. The spectrum of the spherical coating is obviously different from the distribution of the prism and pyramid coating. It not only appears at the value of 1332 ± 2 cm^−1^, and the diamond peak at this value decreases compared with the previous one, the peak width increases, and the peak shift occurs. There is also a significant peak at 1580 cm^−1^, which is lower than the peak at 132 cm^−1^, and the peak width is wider. The reason why the peak width at 1332 ± 2 cm^−1^ is larger than that of the diamond peak is related to the grain size reduction to the nanoscale level, and the significant peak in the range of 1400–1600 cm^−1^ is mainly from the sp^2^ structure of carbon at the grain boundary of the nanodiamond film, so the spherical coating belongs to the nanocrystalline diamond.

### 3.2. Analysis of Material Removal Rate and Surface Roughness in Sapphire Wafer Lapping Using Diamond-Coated Tools

**Figure 10:**
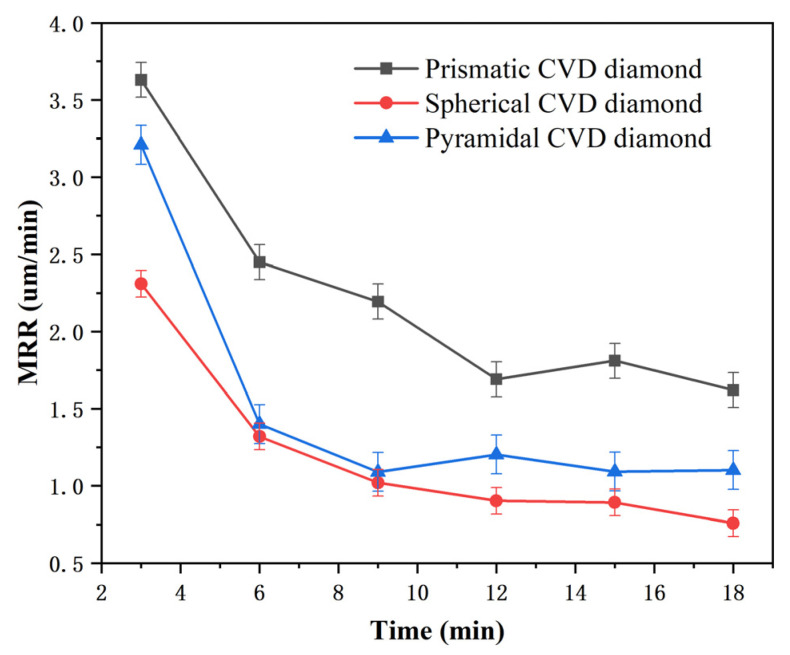
Removal rate of sapphire by lapping with three different morphologies of diamond tools.

**Figure 11:**
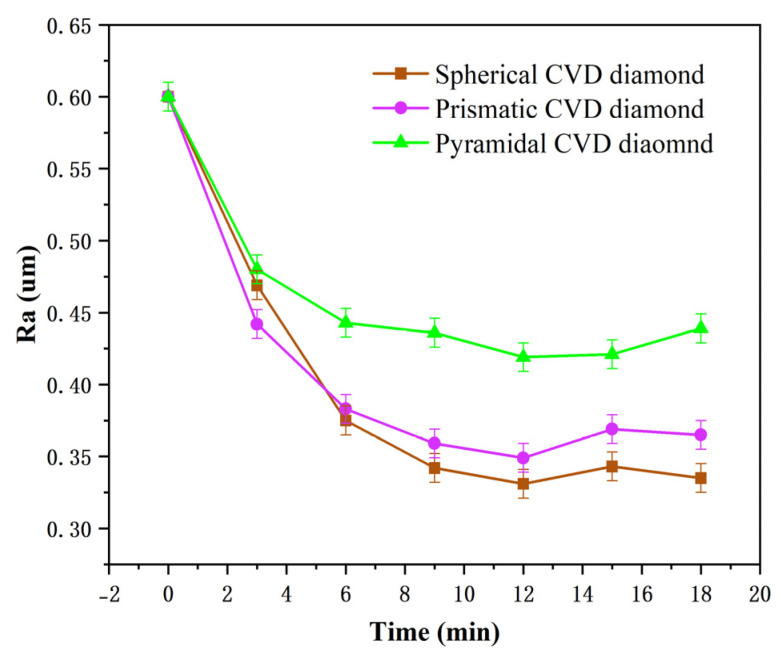
Roughness rate of sapphire by lapping with three different morphologies of diamond tools.

Figure 10 presents the variations in material removal rate and surface roughness over time when using the pyramidal CVD diamond tool. The material removal rate exhibits a decreasing trend with increasing lapping time. The diamond-coated tool contains a large number of sharp crystallites on its surface. The sapphire substrate initially exhibits deep cutting marks and a relatively rough surface. During the lapping process with the CVD diamond grains, the microscopic surface asperities of the sapphire are removed first, resulting in a relatively high material removal rate at the initial stage. When the lapping time reaches 6 min, the material removal rate decreases and stabilizes at approximately 1.20 μm/min. During this stage, the surface waviness of the sapphire substrate is gradually reduced, while the protruding grain structures are progressively fractured. At the same time, debris accumulates on the coating surface, leading to a reduction in the friction coefficient. As a result, the material removal rate continues to decline with the increasing lapping time. As shown in Figure 11, the surface roughness initially decreases with increasing lapping time and subsequently reaches a steady value of approximately 0.44 μm. Within the first 3 min, the roughness of the workpiece surface decreases rapidly from 0.60 to about 0.47 μm. However, when the lapping time reaches 18 min, a slight increase in surface roughness is observed, indicating the emergence of new surface damage.

**Figure 12:**
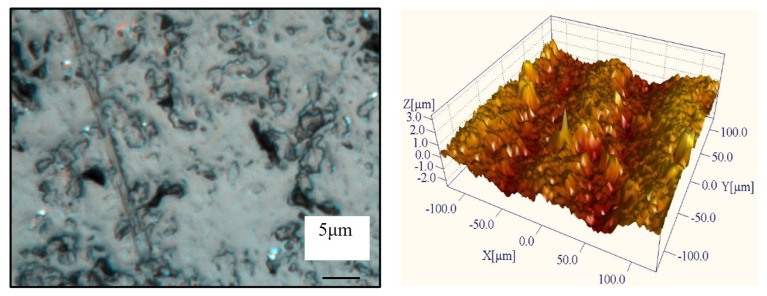
Surface morphology of a pyramidal CVD diamond-coated tool after lapping.

The surface morphology of the sapphire after lapping with the pyramidal CVD diamond tool is shown in Figure 12. The slicing marks generated during wire sawing are almost completely removed, indicating an improvement in the overall surface quality. The mechanism of surface damage evolution is complex. While the high local contact pressure at the sharp tips of the pyramidal diamond crystallites facilitates material removal via micro-fracture (two-body abrasion), it also introduces deep lateral cracks. Furthermore, hard diamond fragments and sapphire debris that are detached during the process can become entrapped at the interface, triggering three-body abrasion which manifests as rolling indentations and random scratches. However, it is worth noting that the triethanolamine in the slurry promotes tribochemical reactions, leading to the formation of a soft hydration layer [31] (e.g., AlO(OH)) on the sapphire surface. This chemically modified layer is easier to remove and helps to mitigate the severity of brittle fractures as the lapping progresses.

As is evident in Figure 11, fresh scratches and surface damage are generated during lapping, and numerous scratches remain across the surface, resulting in a noticeably rough post-lapping finish.

Figure 10 and Figure 11 also illustrate the variations in material removal rate and surface roughness over time when using the prismatic CVD diamond-coated tool. The sharp edges of the prismatic diamond grains provide an effective cutting action on the sapphire surface, thereby accelerating the material removal process. Compared with the pyramidal coating, the prismatic diamond coating provides a larger chip accommodation space, resulting in a slower decline in the material removal rate, which becomes stable at approximately 10 min. When the lapping time reaches around 12 min, the surface roughness attains its minimum value, indicating that most of the cutting-induced damage has been removed. Further lapping leads to the generation of new surface defects, causing the roughness to increase and fluctuate around 0.37 μm.

**Figure 13:**
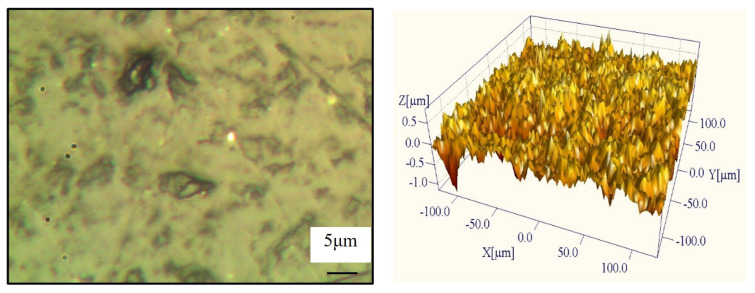
Surface morphology of prismatic CVD diamond-coated tools after lapping.

The surface morphology of the sapphire after lapping with the prismatic diamond-coated tool is shown in Figure 13. Compared with the surface obtained using the pyramidal coating, the overall surface quality is noticeably improved. The cutting-induced damage from the preceding slicing process is largely removed, and the deep scratches are significantly reduced, although fine scratches remain. Nevertheless, the sapphire surface remains relatively rough after lapping.

Figure 10 and Figure 11 also show the variations in material removal rate and surface roughness over time when using the spherical diamond-coated tool. As illustrated in Figure 10, the material removal rate decreases with the increasing lapping time and begins to stabilize at approximately 6 min, with the rate of decline becoming more gradual. Figure 11 indicates that the surface roughness of the sapphire initially decreases but exhibits a slight increase when the lapping time reaches 15 min. Overall, the sapphire surface obtained after lapping with the spherical diamond coating exhibits a relatively low roughness, stabilizing at approximately 0.34 μm.

**Figure 14:**
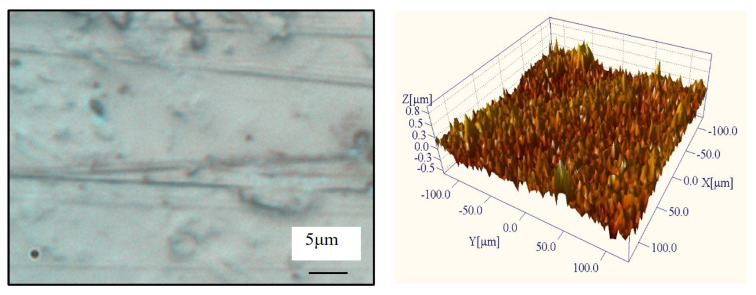
Surface morphology of spherical diamond-coated tool after lapping.

The surface morphology of the sapphire after lapping with the spherical diamond-coated tool is shown in Figure 14. As observed in the figure, smooth and bright regions begin to emerge on the workpiece surface following lapping. Figure 14 also reveals etch pits formed by the chemical action of the lapping slurry, as well as fine scratches that are generated by contact with the spherical diamond grains. Both the scratches and etch pits become smaller and shallower as the process progresses. Compared with the surfaces produced using the pyramidal and prismatic diamond-coated tools, the overall surface quality achieved with the spherical coating is significantly improved.

The thickness of the wear rate on the tool surface calculated after the experiment is shown in Table 2, the average wear depth of the pyramidal CVD diamond-coated tool is 250 Å, and the wear rate is 2.5%. The wear depth of the prismatic CVD diamond-coated tool is 70 Å and the wear rate is 7. The wear depth of the spherical diamond-coated tool is 10 Å and the wear rate is 1%.

Therefore, the use of diamond film-based consolidated tools for lapping sapphire offers distinct advantages. The formation of a CVD diamond film begins with nucleation, followed by oriented crystal growth, during which the interlocking crystallites progressively coalesce to form a continuous film on the substrate surface. During lapping, part of the detached diamond fragments and hard abrasive debris fall into the intergranular gaps and disengage from the contact interface, while the remaining particles act as micro-cutting asperities between the diamond film and the sapphire substrate. Owing to the layer-by-layer growth characteristics of the diamond film, the tool exhibits a degree of self-conditioning, which contributes to the enhanced machining efficiency. Furthermore, the crystallite size of the diamond film can be reduced to the nanometer scale, allowing the surface condition of the tool to be more easily controlled after lapping. These features provide a valuable reference for subsequent precision polishing operations and for the machining of other brittle and hard materials.

## 4. Conclusions

The morphological evolution of CVD diamond films is governed by the balance between carbon deposition and atomic hydrogen etching, rather than simple kinetic energy variations. At higher pressures (3 kPa), the enhanced etching effect of atomic hydrogen effectively suppresses secondary nucleation, fostering the growth of micro-sized grains. Conversely, low atomic hydrogen concentrations at reduced pressures lead to defect accumulation and the formation of cauliflower-like nanocrystalline clusters.

Using the HFCVD method under different deposition conditions, three readily distinguishable CVD diamond coatings with characteristic surface morphologies were fabricated. The spherical coating corresponds to a nanocrystalline diamond film. The pyramidal coating exhibits strong texturing, with the exposed facets being dominated by the (111) plane and an average grain size of approximately 4 μm. The prismatic coating primarily exposes the (100) and (110) crystallographic planes.

In the wet lapping of sapphire, the material removal mechanism is a synergy of mechanical micro-cutting and tribochemical reactions. The prismatic diamond coating achieves the highest thickness removal rate (stabilizing at ~1.7 μm/min), due to the sharp cutting edges of the {100} facets. The spherical coating yields the lowest roughness (Ra ~0.35\mum) but a lower removal rate, which is attributed to the reduced abrasive size and the dominant chemical–mechanical polishing effect induced by the triethanolamine slurry.

## Figures and Tables

**Table 1:** Diamond coating preparation experimental parameters.

Number	Growth Parameters	Pressure (kPa)	Temperature (°C)	CH_4_ Concentration (%)	Gas Flow Rate (SCCM)	Deposition Rates (μm/h)	Depositional Thickness (μm)
1	Atmospheric pressure	1	780	1.5	300	0.09	0.6
2		2	780	1.5	300	0.11	0.65
3		3	780	1.5	300	0.15	0.75
4	Methane content	3	780	1	300	0.06	0.5
5		3	780	2	300	0.15	1.0
6		3	780	3	300	0.2	1.5
7	Substrate temperature	3	730	1.5	300	0.06	0.4
8		3	780	1.5	300	0.10	0.75
9		3	830	1.5	300	0.15	1

**Table 2:** Three types of diamond film tool wear.

Types	Wear Depth (Å)	Wear Rate
pyramidal CVD diamond-coated tool	250	2.5%
prismatic CVD diamond-coated tool	700	7%
spherical diamond-coated tool	100	1%

## Data Availability

The original contributions presented in this study are included in the article. Further inquiries can be directed to the corresponding author.
